# St. Louis Medical and Surgical Journal

**Published:** 1845-04

**Authors:** 


ST. LOUIS MEDICAL AND SURGICAL JOURNAL

We see that our friend, Professor Linton, proposes to enlarge his Journal. We are glad of it. Thus far it has not been what we know he has the power to make it. With ampler room we trust we shall have a more frequent putting forth of those vigorous thoughts which are in him, but which, hitherto, he has kept very much to himself. His third volume will commence with a number of 48 pages in a neat cover. He is to be assisted by Dr. McPheeters, whose talents and devotion to his profession are said to fit him for the task of conducting a journal of medicine. Y.

